# Physicians using ultrasound in Danish emergency departments are mostly summoned specialists

**DOI:** 10.1186/s13049-015-0131-1

**Published:** 2015-07-07

**Authors:** Klaus Nielsen, Johnny Rene Meilstrup Lauridsen, Christian Borbjerg Laursen, Mikkel Brabrand

**Affiliations:** Department of Medicine, Section of Respiratory Medicine, University Hospital Hvidovre, Hvidovre, Denmark; Department of Anaesthesia and Intensive Care, Svendborg Hospital, Svendborg, Denmark; Department of Respiratory Medicine, Odense University Hospital, Odense, Denmark; Department of Emergency Medicine, Sydvestjysk Sygehus, Esbjerg, Denmark

## Abstract

**Background:**

Emergency ultrasound is a relatively new diagnostic discipline. It is used as an extension of the clinical examination and is ideal in the setting of acute illness. The objective of this study was to investigate how many Emergency Departments (EDs) in Denmark have implemented emergency ultrasound. We also wanted to give an idea of how many and which physicians have adopted ultrasound as a diagnostic tool so far.

**Methods:**

The study was a cross-sectional, descriptive, multicenter survey that included all physician staffed EDs in Denmark. An Internet based questionnaire was distributed by e-mail to all heads of department. Those departments who responded that ultrasound was available in their department were included in the second part of the study where all physicians working in the ED were contacted and asked to complete a second questionnaire.

**Results:**

All 28 eligible EDs participated in the first part of the study (Response rate: 100 %). 25 EDs (89 %, 95 % CI: 85-93) had ultrasound equipment available. Questionnaires were distributed to 1,872 physicians in these departments and 561 responded (Response rate: 30 %, 95 % CI: 28-32). Overall 257 (46 %, 95 % CI: 42-50) were users of emergency ultrasound and 304 were non-users (54 %, 95 % CI: 50-58). The largest group with 146 respondents (25 %, 95 % CI: 21-29) were anaesthetists with merely consult duty in the ED. When looking exclusively on physicians with on-call duty in the ED, thus excluding anaesthetists, only 146 (35 %, 95 % CI: 30-40) were users of ultrasound while 269 (65 %, 95 % CI: 60-70) were non-users. There was a considerable difference regarding age, level of training, and medical specialty between users and non-users. Users were mainly anaesthetists and attending physicians from other departments. The majority of non-users were young physicians with on call duty in the ED.

**Conclusions:**

We have found that although almost all Danish EDs have ultrasound equipment available, few physicians working in the ED seem to have adopted the tool. Emergency Ultrasound is mainly performed by specialists who are summoned to the ED in case of severe acute illness and not by those physicians who comprise the backbone of the ED around the clock.

## Background

In several countries, the use of diagnostic ultrasound in the ED has, for many years, been a well-integrated part of the initial investigation of the acute patient. In countries like England, Ireland and the United States, ultrasound diagnostics is an educational element for all physicians who complete training that will lead to a permanent position in an ED [1, 2].

Integrating focused ultrasound diagnostics in the approach to the emergency patient, and the provider performing simple ultrasound examinations, so-called point-of-care ultrasound, has several advantages: the procedure carries no risk for the patient, does not imply radiation, and can be performed immediately at the bedside of the critically ill patient. Point-of-care ultrasound is used as a supplement to or as an integrated part of the clinical examination of the patient. It differs from traditional ultrasound diagnostics performed by radiologists, in the sense that the studies are simple, focused and primarily serve the purpose of answering a specific clinical question [3].

The current North American guidelines for emergency medical ultrasound published by the American College of Emergency Physicians (ACEP) and the international consensus document from the International Federation for Emergency Medicine (IFEM) have classified emergency medical ultrasound in different organ-specific core areas [1, 4]. There is a big difference in how far the research has come within these areas, but within the most well-studied areas, there is solid evidence that emergency medical ultrasound improves the initial diagnostics and treatment and in certain groups of patients may improve outcome while maximizing patient satisfaction with a hospital visit [5–9].

In order to make more physicians in the ED familiar with diagnostic ultrasound and give them suitable skills, it is important that the content and quality of training is formalized and that individual physicians are certified at the end of training. Finally, every ED must be able to provide supervised training for physicians undergoing training in emergency medical ultrasound. This requires all EDs to implement ultrasound and that as many physicians as possible are trained and acquire basic skills in emergency medical ultrasound [1].

Recent European studies that have analysed the availability of ultrasound in community EDs have demonstrated that 52 % and 64 % of French and Italian community EDs respectively have access to ultrasound equipment [10, 11]. To our knowledge, no previous study has focused on the use of ultrasound in Danish EDs. This study will focus on showing how many Danish EDs, until now, have implemented ultrasound and line out how widespread the use of ultrasound is among physicians working in the ED.

## Methods

### The study design

The study was conducted as a cross sectional study using online surveys. The first part of the survey was distributed to the head of department of all Danish EDs and aimed at examining how many departments had implemented emergency medicine ultrasound. The second part of the survey was distributed to physicians with either on-call duty in EDs or physicians who perform medical, surgical or critical care consults there. This part of the survey focused on different aspects of emergency medical ultrasound. Data were collected from October 2012 to September 2013.

### The survey

The survey was web-based. It was developed with the assistance of external experts in survey design and was subsequently pilot tested on 10 physicians.

In order to participate in the survey, the individual ED had to be open 24 h around the clock and have physicians on call at all hours. Only the departments that indicated that ultrasound was available in the ED in the first part of the study, participated in the second part of the study. To increase the response rate, all participants were given the opportunity to take part in a draw for a gift prize sponsored by the Danish Society for Emergency Medicine.

### Data Collection

In the first part of the study, the head of department of all 28 Danish EDs received an e-mail invitation to participate in the survey. The selection of which departments were to be included was based on existing research and by telephone contact with the individual hospitals [12]. The departments that did not respond to the first e-mail, received an e-mail reminder and were then contacted by telephone.

In the second part of the study, an e-mail with a link to the on-line survey was sent to all physicians with either on-call duty in EDs or physicians who perform medical, surgical or critical care consults in the ED.

### Statistics

The collected data were analyzed descriptively and presented as median (5-95 % percentiles) or proportions (95 % confidence interval (CI) calculated using the Clopper-Pearson method).

Data analysis was performed in LimeSurvey (Open Source application, www.limesurvey.org) and SPSS 19 (IBM Corporation, New York, USA).

## Results

The head of department at all 28 EDs responded to the first part of our survey (response rate 100 %). Twenty-five (89 %, 95 % CI: 85-93), responded that their ED had ultrasound equipment available. Twenty-two (79 %, 95 % CI: 71-87), responded that the equipment was physically located in the ED and 19 (68 %, 95 % CI: 56-80) indicated that the equipment was the property of the department itself. In the second part of the study, we contacted physicians in the 25 EDs with ultrasound equipment available. Questionnaires were sent to 1872 physicians and we received 635 responses (34 %, 95 % CI: 32-36) of which 74 had to be excluded because they were incomplete or completed by physicians who did not meet the inclusion criteria. Of the remaining 561 (30 %, 95 % CI: 28-32), physicians representing the department of anaesthesia and intensive care were by far the largest group with a total of 146 respondents (25 %, 95 % CI: 21-29). Overall 257 (46 %, 95 % CI: 42-50) responded that they include ultrasound in their work with patients in the ED while 304 (54 %, 95 % CI: 50-58) did not. However, when looking separately on the group of 415 physicians without a background in anaesthesia and intensive care, only 146 (35 %, 95 % CI: 30-40) of them stated that they used ultrasound in their work in the ED while 269 (65 %, 95 % CI: 60-70) did not.

### Users of ultrasound in the ED

Among users of ultrasound, there was a clear predominance of consultants and physicians in specialist training, respectively, 110 (43 %, 95 % CI: 37-49) and 84 (33 %, 95 % CI: 27-39) who were attending physicians (Table 1). The median age was 38 years (5-95 % percentiles: 27 to 58). It was characteristic of this group as a whole that 136 (53 %, 95 % CI: 47-59) of the physicians did not have on call duty in the ED but were paged when a consult was needed. 59 (23 %, 95 % CI: 18-29) physicians were employed in a different department, but had on call duty in the ED, while 50 (19 %, 95 % CI: 15-25) were employed in the ED.

**Table 1:** Users of ultrasound in the Emergency Department (n = 257)

		**Frequency**	**Percent**	**(95 % CI)**
**Gender**				
	Female	86	33 %	(28-40)
	Male	171	67 %	(60-72)
**Age**				
	Median	38 (5-95 % percentiles: 27,0-58,7)		
**Level of training**				
	Intern	15	6 %	(3-9)
	First year of specialist training	26	10 %	(7-14)
	Second year or later in specialist training	84	33 %	(27-39)
	Consultant	110	43 %	(37-49)
	Other non-specialists	16	6 %	(4-10)
	Medical students	1	0 %	(0-2)
	Other	5	2 %	(1-4)
**Level of responsibility during on call duty**				
	Junior resident	70	27 %	(22-33)
	Senior resident	41	16 %	(12-21)
	Attending physician	146	57 %	(51-63)
**Affiliation with the Emergency Department**				
	Employee of the Emergency Department	50	19 %	(15-25)
	Employee of different department but on call duty in the emergency department	59	23 %	(18-29)
	Employee of different department performing consults in the emergency department	136	53 %	(47-59)
	Other	12	5 %	(3-7)
**Distribution within specialties**				
	Emergency Medicine^a^	5	2 %	(1-4)
	Anaesthesia and Intensive Care	111	43 %	(37-50)
	General Practice	10	4 %	(2-7)
	Internal Medicine	62	24 %	(19-30)
	Surgery	47	18 %	(14-24)
	Other	22	9 %	(5-13)

^a^a medical field but not a medical specialty in Denmark

Looking on the distribution within specialties, the vast majority of users of ultrasound were physicians working in anaesthesia and intensive care, 111 (43 %, 95 % CI: 37-50), followed by physicians working within an internal medicine speciality, 62 (24 %, 95 % CI: 19-30) or surgery, 47 (18 %, 95 % CI: 14-24).

### Non-users of ultrasound in the ED

In the group of non-users we found a more even distribution in terms of level of training, and physicians in this group were younger, with a median age of 33 years (5-95 % percentiles: 27 to 54 (Table 2)). The majority of physicians in this group were either junior residents or senior residents respectively, 131 (43 %, 95 %CI: 38-49) and 70 (23 %, 95 % CI: 18-28). Most were either employees of the ED, 56 (18 %, 95 % CI: 14-23) or employees in a department with on call duty in the ED, 163 (54 %, 95 % CI: 48-59). When the physicians were asked what made them refrain from using ultrasound, 247 (81 %, 95 % CI: 76-86) of them answered that they did not feel competent to perform ultrasound examinations.

**Table 2:** Non-users of ultrasound in the Emergency Department (n = 304)

		**Frequency**	**Percent**	**(95 % CI)**
**Gender**				
	Female	162	53.3 %	(47,5-59.0)
	Male	142	46.7 %	(40,9-52,5)
**Age**				
	Median	33	(5-95 % percentiles: 27,0-54,1)	
**Level of training**				
	Intern	69	22.7 %	(18,1-27,8)
	First year of specialist training	48	15.8 %	(11,8-20,3)
	Second year or later in specialist training	86	28.3 %	(23,3-33,7)
	Consultant	69	22.7 %	(18,1-27,8)
	Other non-specialists	16	5.3 %	(3,0-8,4)
	Medical students	9	3.0 %	(1,4-5,5)
	Other	7	2.3 %	(0,9-4,6)
**Level of responsibility during on call duty**				
	Junior resident	131	43.1 %	(37,5-48,9)
	Senior resident	70	23.0 %	(18,4-28,2)
	Attending physician	103	33.9 %	(28,6-39,5)
**Affiliation with the Emergency Department**				
	Employee of the Emergency Department	56	18.4 %	(14,2-23,2)
	Employee of different department but on call duty in the emergency department	163	53.6 %	(47,8-59,3
	Employee of different department performing consults in the emergency department	66	21.7 %	(17,2-26,8)
	Other	19	6.3 %	(3,8-9,6)

## Discussion

Our results show that most Danish EDs have ultrasound equipment available and that the physicians who use it are typically experienced physicians, mainly specialists and physicians in specialist training who are not on call in the ED but are summoned when a consult or assistance is needed. The vast majority of users consist of anaesthetists who work primarily in surgical operation theatres, recovery wards and intensive care units, who typically are called to the ED when specific summoning criteria are met (Mobile Emergency Team, cardiac arrest or trauma team, *etc.*) [13]. The second largest group of users consists of surgeons and physicians within cardiology and pulmonary medicine, *i.e.* specialties where ultrasound skills, to varying degrees, are part of specialty training.

The overall availability of ultrasound equipment of 89 % in Danish EDs is higher than what has been found in similar studies in other European countries [10, 11]. Our study included only EDs that are open 24 h around the clock and have physicians on-call.

Many smaller Danish hospitals have EDs that have limited opening hours or do not have physicians on-call. The exclusion of the smaller EDs might explain the difference in availability found in our study and similar studies.

The existing knowledge about the staffing of Danish EDs shows that the backbone is largely made up of junior physicians [14] who attend to patients 24 h a day. Our results suggest that it is this group of physicians in particular who do not make use of ultrasound in their work in the ED and the vast majority do not feel qualified to do so.

It is therefore long way to go before we can say that the Danish EDs can provide qualified, focused ultrasound examinations on an international level of acute patients around the clock.

Part of the explanation probably lies in the fact that very few EDs have a larger permanent staff of physicians employed. If this was indeed the case, it would be much easier to provide training, supervision and quality assessment and to ensure that the every physician knows the indications and limitations for focused ultrasound examinations, reaches a high level of imaging competency and learns how to incorporate ultrasound findings in the clinical management of the patient.

In the United States the development of emergency medical ultrasound seems to really have taken off after ACEP published its Emergency Ultrasound Guidelines for the first time in 2001 and again in 2009 [1]. In Denmark, the Danish Society for Emergency Medicine recently published an article proposing a framework for the implementation, education, research and clinical use of ultrasound in Danish EDs and this might, in the future, contribute to a more widespread use of the tool [15].

### Limitations of the study

Any type of survey is affected by bias. The overall response rate for this survey is low, but fully comparable to other similar studies [16]. It is a known fact that web-based surveys often do not achieve response rates that are much higher than 20-40 % [17]. A low response rate will cause a certain degree of non-response bias and means that our data can not be used to accurately illustrate the proportion of physicians in Danish EDs that use ultrasound and the proportion who do not. Furthermore selection bias causes physicians who find the subject of the study interesting more prone to participate in the study.

We assume that non-respondents in our study differ from respondents in the way that they do not make use of ultrasound in their daily clinical work and not consider it relevant. If a greater proportion of non-respondents had participated, it is likely that the group of non-users would have been larger but it would not have changed the distribution of the results within the group of users.

## Conclusions

Emergency medical ultrasound in an international perspective, is a well-integrated part of clinical practice. Our data, however, show some indication that the use of the tool is not very common among the physicians who comprise the backbone of medical staff in Danish EDs. The technological development of ultrasound has resulted in cheaper, better and smaller equipment and the possibilities of emergency medical ultrasound will continue to grow. However, there is a need for research and increased focus on formal training, certification, supervision and quality assurance in the individual EDs.
